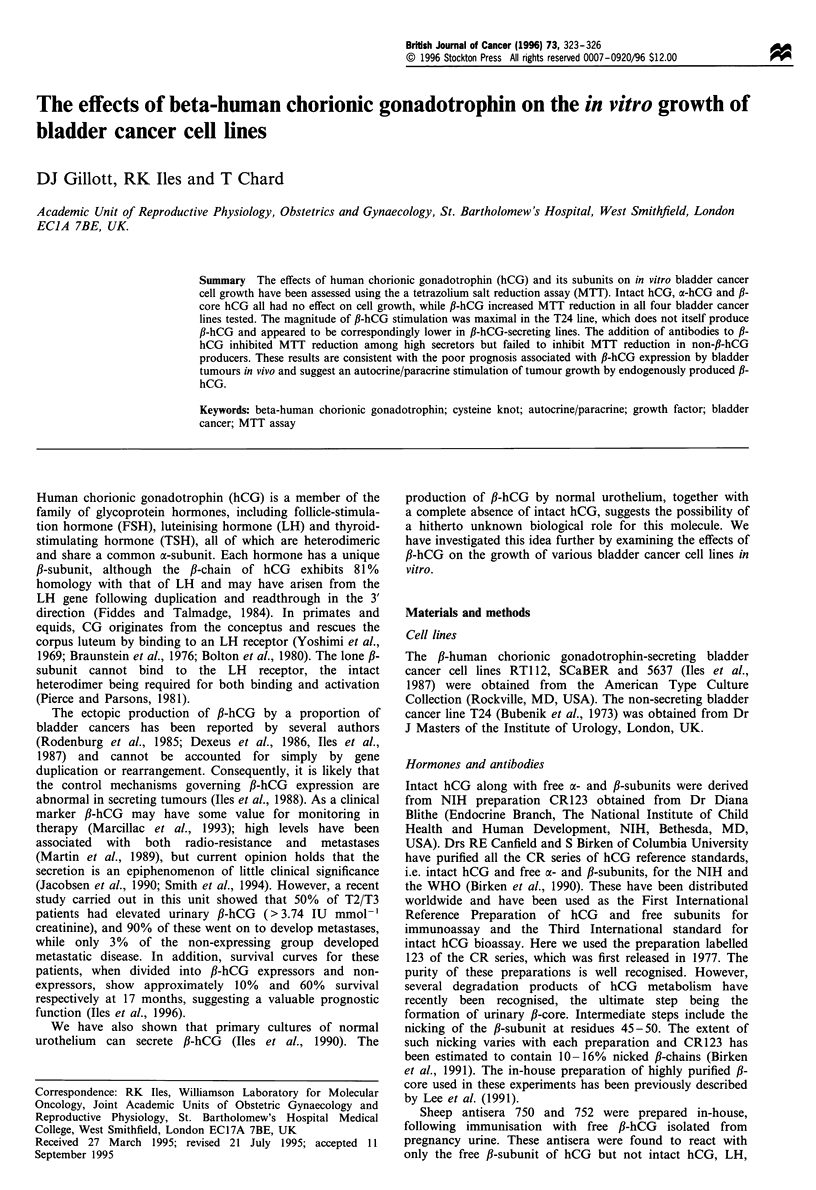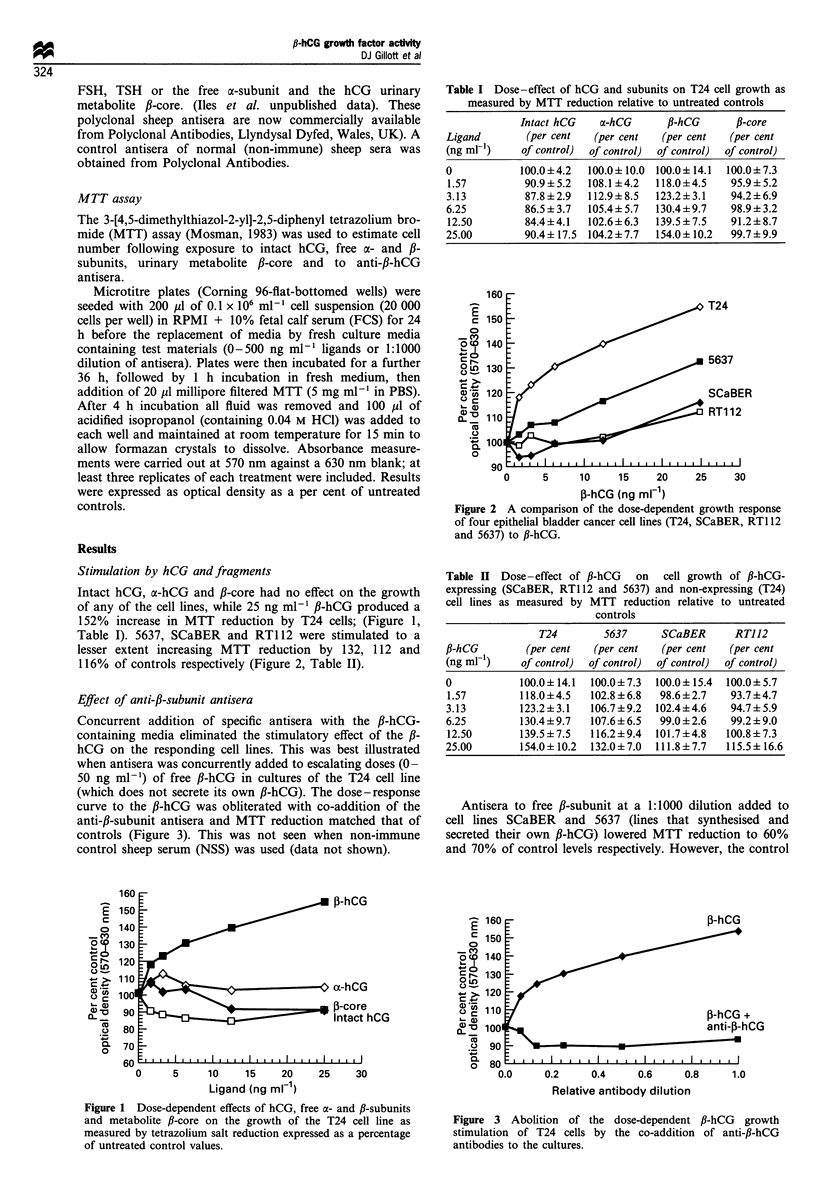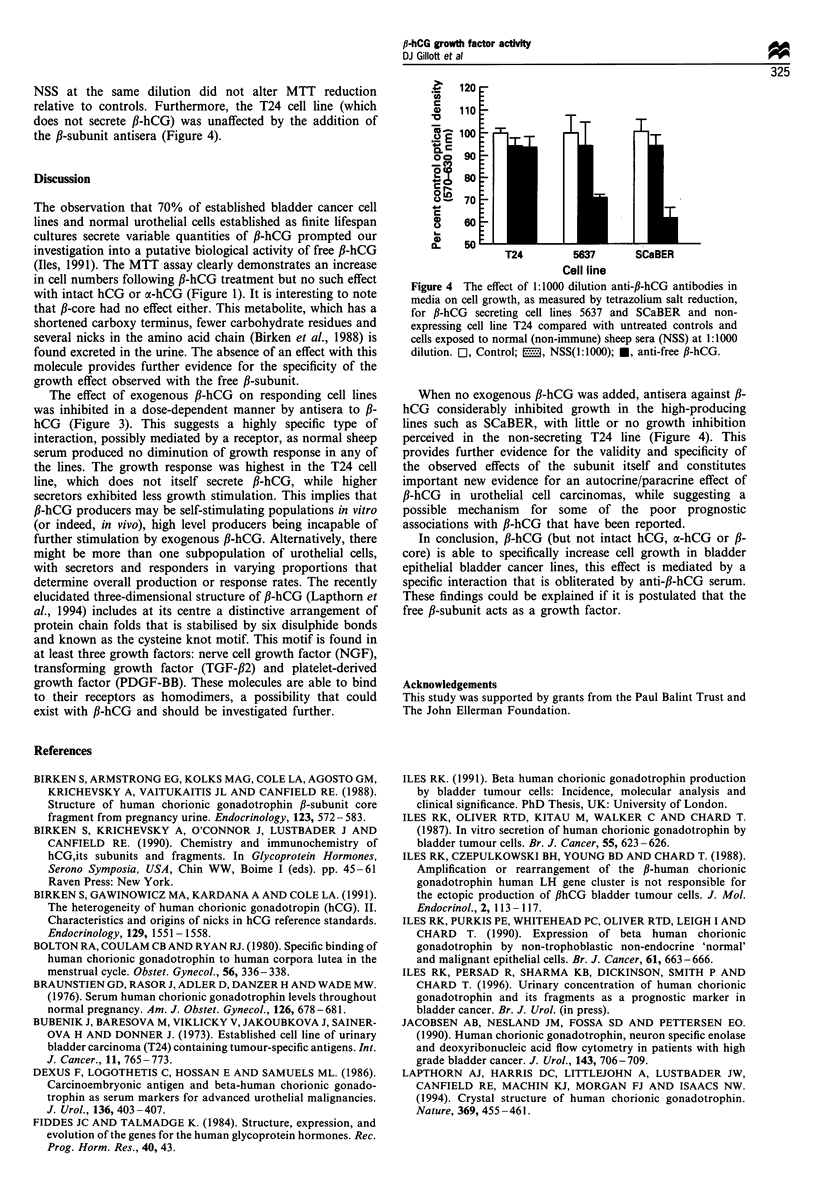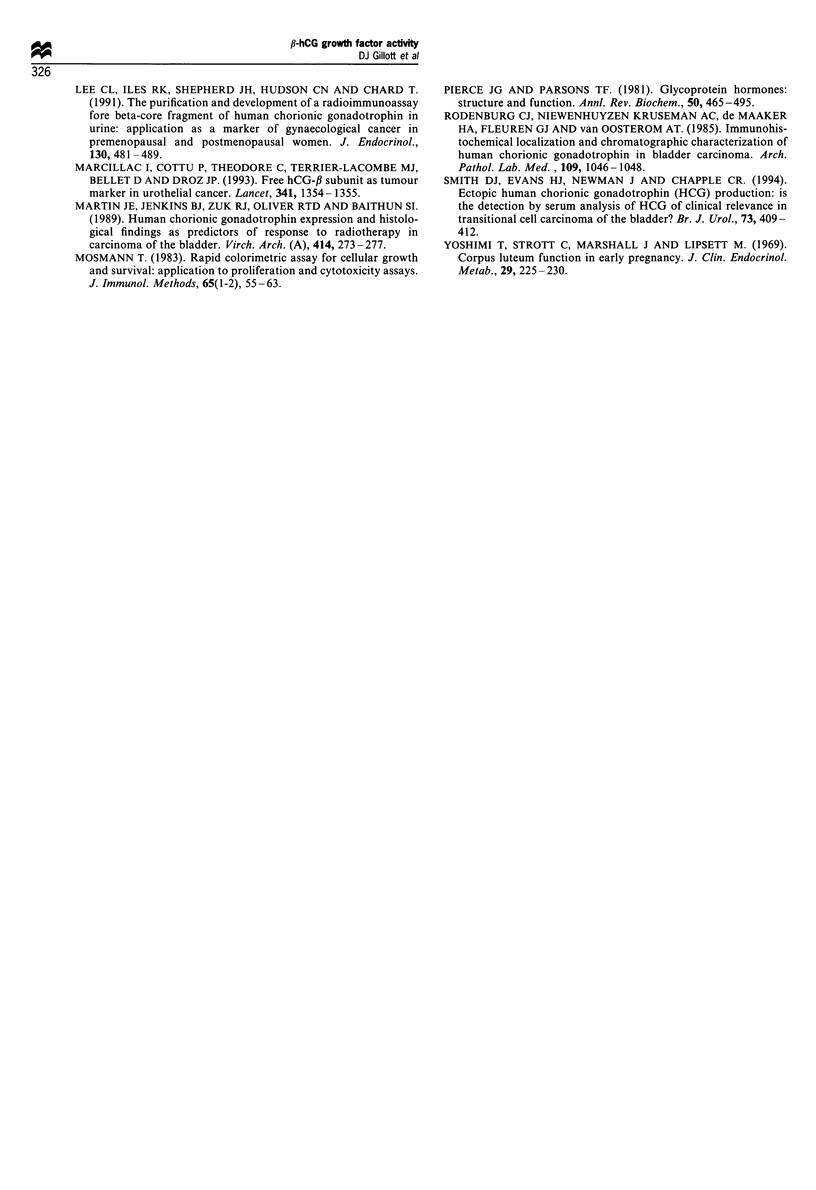# The effects of beta-human chorionic gonadotrophin on the in vitro growth of bladder cancer cell lines.

**DOI:** 10.1038/bjc.1996.56

**Published:** 1996-02

**Authors:** D. J. Gillott, R. K. Iles, T. Chard

**Affiliations:** Academic Unit of Reproductive Physiology, Obstetrics and Gynaecology, St. Bartholomew's Hospital, West Smithfield, London, UK.

## Abstract

The effects of human chorionic gonadotrophin (hCG) and its subunits on in vitro bladder cancer cell growth have been assessed using the a tetrazolium salt reduction assay (MTT). Intact hCG, alpha-hCG and beta-core hCG all had no effect on cell growth, while beta-hCG increased MTT reduction in all four bladder cancer lines tested. The magnitude of beta-hCG stimulation was maximal in the T24 line, which does not itself produce beta-hCG and appeared to be correspondingly lower in beta-hCG-secreting lines. The addition of antibodies to beta-hCG inhibited MTT reduction among high secretors but failed to inhibit MTT reduction in non-beta-hCG producers. These results are consistent with the poor prognosis associated with beta-hCG expression by bladder tumours in vivo and suggest an autocrine/paracrine stimulation of tumour growth by endogenously produced beta-hCG.


British Journal of Cancer (1996) 73, 323-326
© 1996 Stockton Press All rights reserved 0007-0920/96 $12.00

### The effects of beta-human chorionic gonadotrophin on the *in vitro* growth of bladder cancer cell lines

DJ Gillott, RK Iles and T Chard

*Academic Unit of Reproductive Physiology, Obstetrics and Gynaecology, St. Bartholomew's Hospital, West Smithfield, London EC1A 7BE, UK.*

**Summary** The effects of human chorionic gonadotrophin (hCG) and its subunits on *in vitro* bladder cancer cell growth have been assessed using the a tetrazolium salt reduction assay (MTT). Intact hCG, α-hCG and β-core hCG all had no effect on cell growth, while β-hCG increased MTT reduction in all four bladder cancer lines tested. The magnitude of β-hCG stimulation was maximal in the T24 line, which does not itself produce β-hCG and appeared to be correspondingly lower in β-hCG-secreting lines. The addition of antibodies to β-hCG inhibited MTT reduction among high secretors but failed to inhibit MTT reduction in non-β-hCG producers. These results are consistent with the poor prognosis associated with β-hCG expression by bladder tumours *in vivo* and suggest an autocrine/paracrine stimulation of tumour growth by endogenously produced β-hCG.

**Keywords:** beta-human chorionic gonadotrophin; cysteine knot; autocrine/paracrine; growth factor; bladder cancer; MTT assay

Human chorionic gonadotrophin (hCG) is a member of the family of glycoprotein hormones, including follicle-stimulation hormone (FSH), luteinising hormone (LH) and thyroid-stimulating hormone (TSH), all of which are heterodimeric and share a common α-subunit. Each hormone has a unique β-subunit, although the β-chain of hCG exhibits 81% homology with that of LH and may have arisen from the LH gene following duplication and readthrough in the 3′ direction (Fiddes and Talmadge, 1984). In primates and equids, CG originates from the conceptus and rescues the corpus luteum by binding to an LH receptor (Yoshimi *et al.*, 1969; Braunstein *et al.*, 1976; Bolton *et al.*, 1980). The lone β-subunit cannot bind to the LH receptor, the intact heterodimer being required for both binding and activation (Pierce and Parsons, 1981).

The ectopic production of β-hCG by a proportion of bladder cancers has been reported by several authors (Rodenburg *et al.*, 1985; Dexeus *et al.*, 1986, Iles *et al.*, 1987) and cannot be accounted for simply by gene duplication or rearrangement. Consequently, it is likely that the control mechanisms governing β-hCG expression are abnormal in secreting tumours (Iles *et al.*, 1988). As a clinical marker β-hCG may have some value for monitoring in therapy (Marcillac *et al.*, 1993); high levels have been associated with both radio-resistance and metastases (Martin *et al.*, 1989), but current opinion holds that the secretion is an epiphenomenon of little clinical significance (Jacobsen *et al.*, 1990; Smith *et al.*, 1994). However, a recent study carried out in this unit showed that 50% of T2/T3 patients had elevated urinary β-hCG (>3.74 IU $mmol^{-1}$ creatinine), and 90% of these went on to develop metastases, while only 3% of the non-expressing group developed metastatic disease. In addition, survival curves for these patients, when divided into β-hCG expressors and non-expressors, show approximately 10% and 60% survival respectively at 17 months, suggesting a valuable prognostic function (Iles *et al.*, 1996).

We have also shown that primary cultures of normal urothelium can secrete β-hCG (Iles *et al.*, 1990). The production of β-hCG by normal urothelium, together with a complete absence of intact hCG, suggests the possibility of a hitherto unknown biological role for this molecule. We have investigated this idea further by examining the effects of β-hCG on the growth of various bladder cancer cell lines *in vitro*.

Correspondence: RK Iles, Williamson Laboratory for Molecular Oncology, Joint Academic Units of Obstetric Gynaecology and Reproductive Physiology, St. Bartholomew's Hospital Medical College, West Smithfield, London EC17A 7BE, UK
Received 27 March 1995; revised 21 July 1995; accepted 11 September 1995

### Materials and methods

### *Cell lines*

The β-human chorionic gonadotrophin-secreting bladder cancer cell lines RT112, SCaBER and 5637 (Iles *et al.*, 1987) were obtained from the American Type Culture Collection (Rockville, MD, USA). The non-secreting bladder cancer line T24 (Bubenik *et al.*, 1973) was obtained from Dr J Masters of the Institute of Urology, London, UK.

### *Hormones and antibodies*

Intact hCG along with free α- and β-subunits were derived from NIH preparation CR123 obtained from Dr Diana Blithe (Endocrine Branch, The National Institute of Child Health and Human Development, NIH, Bethesda, MD, USA). Drs RE Canfield and S Birken of Columbia University have purified all the CR series of hCG reference standards, i.e. intact hCG and free α- and β-subunits, for the NIH and the WHO (Birken *et al.*, 1990). These have been distributed worldwide and have been used as the First International Reference Preparation of hCG and free subunits for immunoassay and the Third International standard for intact hCG bioassay. Here we used the preparation labelled 123 of the CR series, which was first released in 1977. The purity of these preparations is well recognised. However, several degradation products of hCG metabolism have recently been recognised, the ultimate step being the formation of urinary β-core. Intermediate steps include the nicking of the β-subunit at residues 45-50. The extent of such nicking varies with each preparation and CR123 has been estimated to contain 10-16% nicked β-chains (Birken *et al.*, 1991). The in-house preparation of highly purified β-core used in these experiments has been previously described by Lee *et al.* (1991).

Sheep antisera 750 and 752 were prepared in-house, following immunisation with free β-hCG isolated from pregnancy urine. These antisera were found to react with only the free β-subunit of hCG but not intact hCG, LH,

FSH, TSH or the free α-subunit and the hCG urinary metabolite β-core. (Iles *et al.* unpublished data). These polyclonal sheep antisera are now commercially available from Polyclonal Antibodies, Llyndysal Dyfed, Wales, UK). A control antisera of normal (non-immune) sheep sera was obtained from Polyclonal Antibodies.

*MTT assay*

The 3-[4,5-dimethylthiazol-2-yl]-2,5-diphenyl tetrazolium bromide (MTT) assay (Mosman, 1983) was used to estimate cell number following exposure to intact hCG, free α- and β-subunits, urinary metabolite β-core and to anti-β-hCG antisera.

Microtitre plates (Corning 96-flat-bottomed wells) were seeded with 200 μl of $0.1 \times 10^6$ $ml^{-1}$ cell suspension (20 000 cells per well) in RPMI + 10% fetal calf serum (FCS) for 24 h before the replacement of media by fresh culture media containing test materials (0–500 ng $ml^{-1}$ ligands or 1:1000 dilution of antisera). Plates were then incubated for a further 36 h, followed by 1 h incubation in fresh medium, then addition of 20 μl millipore filtered MTT (5 mg $ml^{-1}$ in PBS). After 4 h incubation all fluid was removed and 100 μl of acidified isopropanol (containing 0.04 M HCl) was added to each well and maintained at room temperature for 15 min to allow formazan crystals to dissolve. Absorbance measurements were carried out at 570 nm against a 630 nm blank; at least three replicates of each treatment were included. Results were expressed as optical density as a per cent of untreated controls.

### Results

*Stimulation by hCG and fragments*

Intact hCG, α-hCG and β-core had no effect on the growth of any of the cell lines, while 25 ng $ml^{-1}$ β-hCG produced a 152% increase in MTT reduction by T24 cells; (Figure 1, Table I). 5637, SCaBER and RT112 were stimulated to a lesser extent increasing MTT reduction by 132, 112 and 116% of controls respectively (Figure 2, Table II).

*Effect of anti-β-subunit antisera*

Concurrent addition of specific antisera with the β-hCG-containing media eliminated the stimulatory effect of the β-hCG on the responding cell lines. This was best illustrated when antisera was concurrently added to escalating doses (0–50 ng $ml^{-1}$) of free β-hCG in cultures of the T24 cell line (which does not secrete its own β-hCG). The dose–response curve to the β-hCG was obliterated with co-addition of the anti-β-subunit antisera and MTT reduction matched that of controls (Figure 3). This was not seen when non-immune control sheep serum (NSS) was used (data not shown).

**Table I** Dose–effect of hCG and subunits on T24 cell growth as measured by MTT reduction relative to untreated controls

| Ligand (ng $ml^{-1}$) | *Intact hCG (per cent of control)* | *α-hCG (per cent of control)* | *β-hCG (per cent of control)* | *β-core (per cent of control)* |
|---|---|---|---|---|
| 0 | 100.0±4.2 | 100.0±10.0 | 100.0±14.1 | 100.0±7.3 |
| 1.57 | 90.9±5.2 | 108.1±4.2 | 118.0±4.5 | 95.9±5.2 |
| 3.13 | 87.8±2.9 | 112.9±8.5 | 123.2±3.1 | 94.2±6.9 |
| 6.25 | 86.5±3.7 | 105.4±5.7 | 130.4±9.7 | 98.9±3.2 |
| 12.50 | 84.4±4.1 | 102.6±6.3 | 139.5±7.5 | 91.2±8.7 |
| 25.00 | 90.4±17.5 | 104.2±7.7 | 154.0±10.2 | 99.7±9.9 |

**Figure 2** A comparison of the dose-dependent growth response of four epithelial bladder cancer cell lines (T24, SCaBER, RT112 and 5637) to β-hCG.

**Table II** Dose–effect of β-hCG on cell growth of β-hCG-expressing (SCaBER, RT112 and 5637) and non-expressing (T24) cell lines as measured by MTT reduction relative to untreated controls

| β-hCG (ng $ml^{-1}$) | *T24 (per cent of control)* | *5637 (per cent of control)* | *SCaBER (per cent of control)* | *RT112 (per cent of control)* |
|---|---|---|---|---|
| 0 | 100.0±14.1 | 100.0±7.3 | 100.0±15.4 | 100.0±5.7 |
| 1.57 | 118.0±4.5 | 102.8±6.8 | 98.6±2.7 | 93.7±4.7 |
| 3.13 | 123.2±3.1 | 106.7±9.2 | 102.4±4.6 | 94.7±5.9 |
| 6.25 | 130.4±9.7 | 107.6±6.5 | 99.0±2.6 | 99.2±9.0 |
| 12.50 | 139.5±7.5 | 116.2±9.4 | 101.7±4.8 | 100.8±7.3 |
| 25.00 | 154.0±10.2 | 132.0±7.0 | 111.8±7.7 | 115.5±16.6 |

Antisera to free β-subunit at a 1:1000 dilution added to cell lines SCaBER and 5637 (lines that synthesised and secreted their own β-hCG) lowered MTT reduction to 60% and 70% of control levels respectively. However, the control

**Figure 1** Dose-dependent effects of hCG, free α- and β-subunits and metabolite β-core on the growth of the T24 cell line as measured by tetrazolium salt reduction expressed as a percentage of untreated control values.

**Figure 3** Abolition of the dose-dependent β-hCG growth stimulation of T24 cells by the co-addition of anti-β-hCG antibodies to the cultures.

NSS at the same dilution did not alter MTT reduction relative to controls. Furthermore, the T24 cell line (which does not secrete β-hCG) was unaffected by the addition of the β-subunit antisera (Figure 4).

### Discussion

The observation that 70% of established bladder cancer cell lines and normal urothelial cells established as finite lifespan cultures secrete variable quantities of β-hCG prompted our investigation into a putative biological activity of free β-hCG (Iles, 1991). The MTT assay clearly demonstrates an increase in cell numbers following β-hCG treatment but no such effect with intact hCG or α-hCG (Figure 1). It is interesting to note that β-core had no effect either. This metabolite, which has a shortened carboxy terminus, fewer carbohydrate residues and several nicks in the amino acid chain (Birken *et al.*, 1988) is found excreted in the urine. The absence of an effect with this molecule provides further evidence for the specificity of the growth effect observed with the free β-subunit.

The effect of exogenous β-hCG on responding cell lines was inhibited in a dose-dependent manner by antisera to β-hCG (Figure 3). This suggests a highly specific type of interaction, possibly mediated by a receptor, as normal sheep serum produced no diminution of growth response in any of the lines. The growth response was highest in the T24 cell line, which does not itself secrete β-hCG, while higher secretors exhibited less growth stimulation. This implies that β-hCG producers may be self-stimulating populations *in vitro* (or indeed, *in vivo*), high level producers being incapable of further stimulation by exogenous β-hCG. Alternatively, there might be more than one subpopulation of urothelial cells, with secretors and responders in varying proportions that determine overall production or response rates. The recently elucidated three-dimensional structure of β-hCG (Lapthorn *et al.*, 1994) includes at its centre a distinctive arrangement of protein chain folds that is stabilised by six disulphide bonds and known as the cysteine knot motif. This motif is found in at least three growth factors: nerve cell growth factor (NGF), transforming growth factor (TGF-β2) and platelet-derived growth factor (PDGF-BB). These molecules are able to bind to their receptors as homodimers, a possibility that could exist with β-hCG and should be investigated further.

**Figure 4** The effect of 1:1000 dilution anti-β-hCG antibodies in media on cell growth, as measured by tetrazolium salt reduction, for β-hCG secreting cell lines 5637 and SCaBER and non-expressing cell line T24 compared with untreated controls and cells exposed to normal (non-immune) sheep sera (NSS) at 1:1000 dilution. □, Control; ▒, NSS(1:1000); ■, anti-free β-hCG.

When no exogenous β-hCG was added, antisera against β-hCG considerably inhibited growth in the high-producing lines such as SCaBER, with little or no growth inhibition perceived in the non-secreting T24 line (Figure 4). This provides further evidence for the validity and specificity of the observed effects of the subunit itself and constitutes important new evidence for an autocrine/paracrine effect of β-hCG in urothelial cell carcinomas, while suggesting a possible mechanism for some of the poor prognostic associations with β-hCG that have been reported.

In conclusion, β-hCG (but not intact hCG, α-hCG or β-core) is able to specifically increase cell growth in bladder epithelial bladder cancer lines, this effect is mediated by a specific interaction that is obliterated by anti-β-hCG serum. These findings could be explained if it is postulated that the free β-subunit acts as a growth factor.

**Acknowledgements**
This study was supported by grants from the Paul Balint Trust and The John Ellerman Foundation.

### References

BIRKEN S, ARMSTRONG EG, KOLKS MAG, COLE LA, AGOSTO GM, KRICHEVSKY A, VAITUKAITIS JL AND CANFIELD RE. (1988). Structure of human chorionic gonadotrophin β-subunit core fragment from pregnancy urine. *Endocrinology*, **123,** 572–583.

BIRKEN S, KRICHEVSKY A, O'CONNOR J, LUSTBADER J AND CANFIELD RE. (1990). Chemistry and immunochemistry of hCG,its subunits and fragments. In *Glycoprotein Hormones, Serono Symposia, USA*, Chin WW, Boime I (eds). pp. 45–61 Raven Press: New York.

BIRKEN S, GAWINOWICZ MA, KARDANA A AND COLE LA. (1991). The heterogeneity of human chorionic gonadotropin (hCG). II. Characteristics and origins of nicks in hCG reference standards. *Endocrinology*, **129,** 1551–1558.

BOLTON RA, COULAM CB AND RYAN RJ. (1980). Specific binding of human chorionic gonadotrophin to human corpora lutea in the menstrual cycle. *Obstet. Gynecol.*, **56,** 336–338.

BRAUNSTIEN GD, RASOR J, ADLER D, DANZER H AND WADE MW. (1976). Serum human chorionic gonadotrophin levels throughout normal pregnancy. *Am. J. Obstet. Gynecol.*, **126,** 678–681.

BUBENIK J, BARESOVA M, VIKLICKY V, JAKOUBKOVA J, SAINEROVA H AND DONNER J. (1973). Established cell line of urinary bladder carcinoma (T24) containing tumour-specific antigens. *Int. J. Cancer.*, **11,** 765–773.

DEXUS F, LOGOTHETIS C, HOSSAN E AND SAMUELS ML. (1986). Carcinoembryonic antigen and beta-human chorionic gonadotrophin as serum markers for advanced urothelial malignancies. *J. Urol.*, **136,** 403–407.

FIDDES JC AND TALMADGE K. (1984). Structure, expression, and evolution of the genes for the human glycoprotein hormones. *Rec. Prog. Horm. Res.*, **40,** 43.

ILES RK. (1991). Beta human chorionic gonadotrophin production by bladder tumour cells: Incidence, molecular analysis and clinical significance. PhD Thesis, UK: University of London.

ILES RK, OLIVER RTD, KITAU M, WALKER C AND CHARD T. (1987). In vitro secretion of human chorionic gonadotrophin by bladder tumour cells. *Br. J. Cancer*, **55,** 623–626.

ILES RK, CZEPULKOWSKI BH, YOUNG BD AND CHARD T. (1988). Amplification or rearrangement of the β-human chorionic gonadotrophin human LH gene cluster is not responsible for the ectopic production of βhCG bladder tumour cells. *J. Mol. Endocrinol.*, **2,** 113–117.

ILES RK, PURKIS PE, WHITEHEAD PC, OLIVER RTD, LEIGH I AND CHARD T. (1990). Expression of beta human chorionic gonadotrophin by non-trophoblastic non-endocrine 'normal' and malignant epithelial cells. *Br. J. Cancer*, **61,** 663–666.

ILES RK, PERSAD R, SHARMA KB, DICKINSON, SMITH P AND CHARD T. (1996). Urinary concentration of human chorionic gonadotrophin and its fragments as a prognostic marker in bladder cancer. *Br. J. Urol.* (in press).

JACOBSEN AB, NESLAND JM, FOSSA SD AND PETTERSEN EO. (1990). Human chorionic gonadotrophin, neuron specific enolase and deoxyribonucleic acid flow cytometry in patients with high grade bladder cancer. *J. Urol.*, **143,** 706–709.

LAPTHORN AJ, HARRIS DC, LITTLEJOHN A, LUSTBADER JW, CANFIELD RE, MACHIN KJ, MORGAN FJ AND ISAACS NW. (1994). Crystal structure of human chorionic gonadotrophin. *Nature*, **369,** 455–461.

LEE CL, ILES RK, SHEPHERD JH, HUDSON CN AND CHARD T. (1991). The purification and development of a radioimmunoassay fore beta-core fragment of human chorionic gonadotrophin in urine: application as a marker of gynaecological cancer in premenopausal and postmenopausal women. *J. Endocrinol.*, **130**, 481–489.

MARCILLAC I, COTTU P, THEODORE C, TERRIER-LACOMBE MJ, BELLET D AND DROZ JP. (1993). Free hCG-β subunit as tumour marker in urothelial cancer. *Lancet*, **341**, 1354–1355.

MARTIN JE, JENKINS BJ, ZUK RJ, OLIVER RTD AND BAITHUN SI. (1989). Human chorionic gonadotrophin expression and histological findings as predictors of response to radiotherapy in carcinoma of the bladder. *Virch. Arch.* (A), **414**, 273–277.

MOSMANN T. (1983). Rapid colorimetric assay for cellular growth and survival: application to proliferation and cytotoxicity assays. *J. Immunol. Methods*, **65**(1-2), 55–63.

PIERCE JG AND PARSONS TF. (1981). Glycoprotein hormones: structure and function. *Annl. Rev. Biochem.*, **50**, 465–495.

RODENBURG CJ, NIEWENHUYZEN KRUSEMAN AC, de MAAKER HA, FLEUREN GJ AND van OOSTEROM AT. (1985). Immunohistochemical localization and chromatographic characterization of human chorionic gonadotrophin in bladder carcinoma. *Arch. Pathol. Lab. Med.*, **109**, 1046–1048.

SMITH DJ, EVANS HJ, NEWMAN J AND CHAPPLE CR. (1994). Ectopic human chorionic gonadotrophin (HCG) production: is the detection by serum analysis of HCG of clinical relevance in transitional cell carcinoma of the bladder? *Br. J. Urol.*, **73**, 409–412.

YOSHIMI T, STROTT C, MARSHALL J AND LIPSETT M. (1969). Corpus luteum function in early pregnancy. *J. Clin. Endocrinol. Metab.*, **29**, 225–230.